# Antiviral Mechanism of Action of Epigallocatechin-3-*O*-gallate and Its Fatty Acid Esters

**DOI:** 10.3390/molecules23102475

**Published:** 2018-09-27

**Authors:** Kunihiro Kaihatsu, Miyuki Yamabe, Yasuhito Ebara

**Affiliations:** Graduate School of Human Development and Environment, Kobe University, 3-11 Tsurukabuto, Kobe, Hyogo 657-8501, Japan; 144d420d@stu.kobe-u.ac.jp (M.Y.); ebara@kobe-u.ac.jp (Y.E.)

**Keywords:** catechin, epigallocatechin-3-*O*-gallate, virus inhibition, attachment, entry, fusion, replication, budding, fatty acid derivative

## Abstract

Epigallocatechin-3-*O*-gallate (EGCG) is the major catechin component of green tea (*Cameria sinensis*), and is known to possess antiviral activities against a wide range of DNA viruses and RNA viruses. However, few studies have examined chemical modifications of EGCG in terms of enhanced antiviral efficacy. This paper discusses which steps of virus infection EGCG interferes with, citing previous reports. EGCG appears most likely to inhibits the early stage of infections, such as attachment, entry, and membrane fusion, by interfering with viral membrane proteins. According to the relationships between structure and antiviral activity of catechin derivatives, the 3-galloyl and 5′-OH group of catechin derivatives appear critical to antiviral activities. Enhancing the binding affinity of EGCG to virus particles would thus be important to increase virucidal activity. We propose a newly developed EGCG-fatty acid derivative in which the fatty acid on the phenolic hydroxyl group would be expected to increase viral and cellular membrane permeability. EGCG-fatty acid monoesters showed improved antiviral activities against different types of viruses, probably due to their increased affinity for virus and cellular membranes. Our study promotes the application of EGCG-fatty acid derivatives for the prevention and treatment of viral infections.

## 1. Introduction

### Catechins in Green Tea Extracts and Their Antiviral Activities

**Figure 1:**
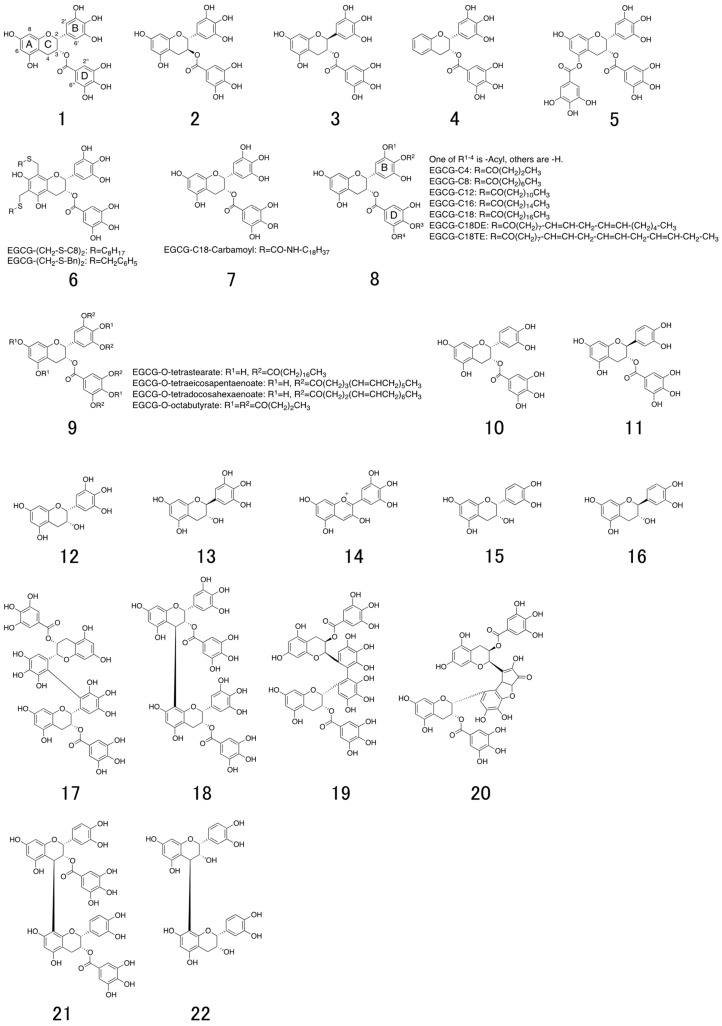

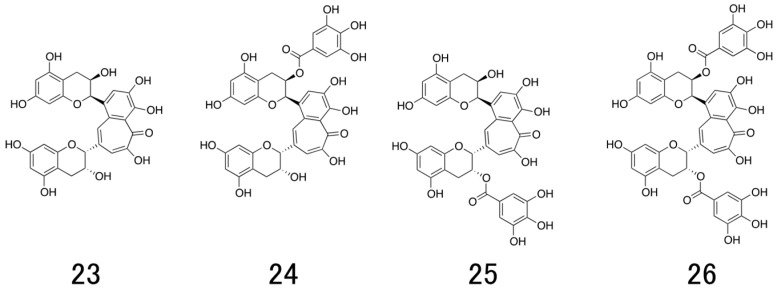
Chemical structures of natural catechins. (−)-epigallocatechin-3-*O*-gallate (EGCG; **1**), (+)-gallocatechin-3-*O*-gallate ((+)-GCG; **2**), (-)-gallocatechin-3-*O*-gallate ((−)-GCG; **3**), 5,7-dideoxy-EGCG (DO-EGCG; **4**), epigallocatechin 3,5-digallate (EGCDG; **5**), EGCG-thioether derivatives (**6**), EGCG-n-octadecylisocyanate derivative (**7**), EGCG-fatty acid monoester derivatives (**8**), EGCG-fatty acid tetra, octaester derivatives (**9**), (−)-epicatechin-3-*O*-gallate (ECG; **10**), (−)-catechin-3-*O*-gallate (CG; **11**), (−)-epigallocatechin (EGC; **12**), (−)-gallocatechin (GC; **13**), delphinidin (**14**), (−)-epicatechin (EC; **15**), (−)-catechin (C; **16**), 2′,2′-bisepigallocatechin digallate (bEGCdG; **17**), rhodisin (**18**), theasinensin A (**19**), P2 (**20**), epicatechin-3-*O*-gallate-(4β→8)-epicatechin-3-*O*-gallate (**21**), procyanidin B2 (**22**), theaflavin (TF; **23**) theaflavin-3-gallate (TF-3-G; **24**), theaflavin-3′-gallate (TF-3′-G; **25**), and theaflavin-3,3′-*O*-digallate (TFDG; **26**).

A series of catechin derivatives are present in green tea extract, representing 30–42% of the total dry weight of tea leaves [1]. The major green tea catechins are (−)-epigallocatechin-3-*O*-gallate (EGCG; **1**), (−)-epigallocatechin (EGC; **12**), (−)-epicatechin (EC; **15**), and (−)-catechin (C; **16**) (Figure 1). In the fermentation process for making black tea, catechins are oxidatively polymerized by polyphenol oxidase and converted to theaflavin (TF; **23**) and theaflavin-3,3′-*O*-digallate (TFDG; **26**). Among these catechins, EGCG is the most abundant and has received much attention because of its biological activities (such as antiviral [2], antimicrobial [3], and anticancer [4] activities) that are superior to those of other catechin derivatives. In this review, we summarize previous research reports regarding the antiviral activity of EGCG and its derivatives in terms of their structures and virus inhibitory mechanisms.

Regarding these biological properties, EGCG (**1**) has been reported to possess a broad spectrum of antiviral activities against DNA viruses such as herpes simplex virus (HSV; *Herpesviridae*) [5,6,7,8,9,10,11], adenovirus (*Adenoviridae*) [10,12], human papilloma virus (HPV; *Papovaviridae*) [13], and hepatitis B virus (HBV; *Hepadnaviridae*) [14], and against (+)-RNA viruses such as hepatitis C virus (HCV; *Flaviviridae*) [10,15,16,17,18,19], Zika virus (ZIKV; *Flaviviridae*) [20,21,22,23], dengue virus (DENV; *Flaviviridae*) [23,24], West Nile viruses (WNV; *Flaviviridae*) [23], Chikungunya virus (CHIKV; *Togaviridae*) [25], and Porcine Reproductive and Respiratory virus (PRRS; *Atteriviridae*) [26], and (−)-RNA viruses such as human immunodeficiency virus (HIV; *Retroviridae*) [27,28,29,30,31,32,33,34,35,36], Ebola virus (EBOV; *Filoviridae*) [37] and influenza virus (*Orthomyxoviridae*) [2,10,38,39,40,41,42,43,44,45,46,47,48].

**Figure 2:**
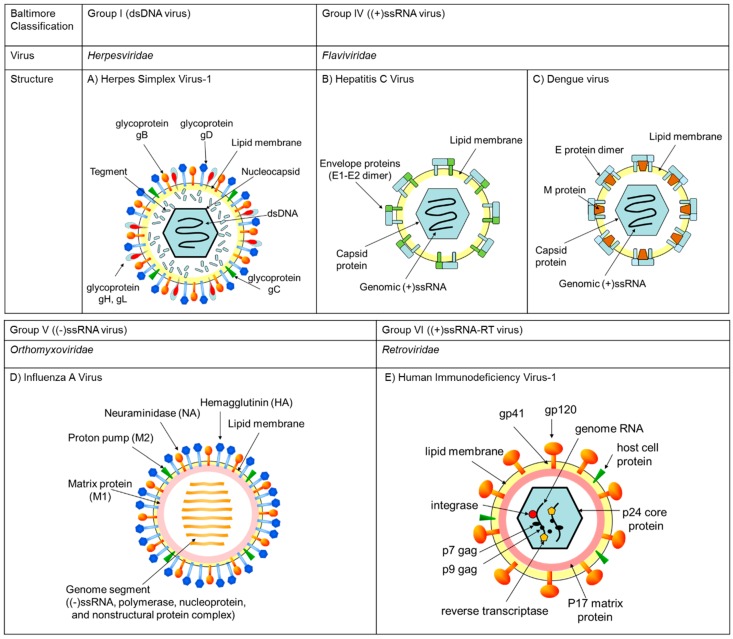
The DNA and RNA viruses described in this study were classified by Baltimore group. A representative of each virus family and their structures are summarized in the figures. (**A**) Herpes simplex virus-1 is a member of *Herpesviridae*, classified to Baltimore group I possessing dsDNA as the genome in a nucleocapsid core enveloped by lipid membrane. (**B**) Hepatitis C virus and (**C**) Dengue virus are member of *Flaviviridae*, classified to Baltimore group IV possessing a (+) single-stranded RNA genome in the viral particle. (**D**) Influenza A virus is a member of *Orthomyxoviridae*, classified to Baltimore group V possessing eight (−)-strand viral RNA genomes in the viral particle. (**E**) Human immunodeficiency virus-1 is a member of *Retroviridae*, classified to Baltimore VI possessing two (+)-strand RNA genomes in a protein core in the viral particle.

**Figure 3:**
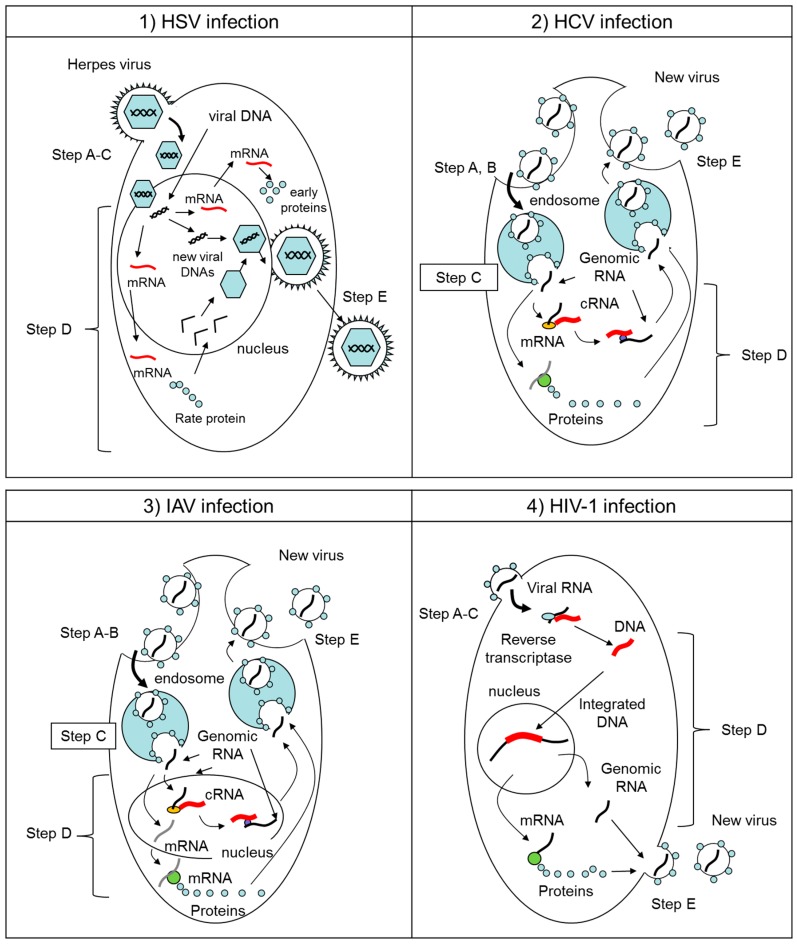
Schematic overviews of the virus life cycles of (**1**) HSV, (**2**) HCV, (**3**) IAV, and (**4**) HIV-1 in infected cells. Their infection processes were divided by five steps. Step A: virus attaches to cell surface receptor. Step B: virus entry into cells by endocytosis. Step C: virus-cell membrane fusion. Step D: viral genome replication and synthesis of progeny viral components. Step E: budding of newly developed progeny virions.

In this paper, we first briefly introduce the classifications (Figure 2), structures (Figure 2), and life cycles of viruses (Figure 3) in Section 2. Second, in Section 3, Section 4 and Section 5 we identify those steps of virus infection that EGCG (**1**) interferes with, according to the literature. Third, in Section 6 we discuss the relationships between structure and anti-viral activity for catechin derivatives. Last, we introduce in Section 7 previously reported lipophilic EGCG derivatives and propose in Section 8 a novel type of EGCG-fatty acid derivative **8** that possesses potent antiviral activity.

## 2. Classifications, Structures, and Life Cycles of Viruses Discussed in This Review

### 2.1. Herpes Simplex Virus (HSV)

Herpes simplex virus type 1 (HSV-1) and type 2 (HSV-2) belong to the *Herpesviridae* family and are known as common pathogens that cause localized skin infections of the mucosal epithelia of the genitals, oral cavity, pharynx, esophagus and eyes. They have a complex structure, comprising an icosahedral, double-stranded DNA-containing capsid, located within the virion and surrounded by a membrane envelope heterogeneously studded with morphologically distinct spikes formed by 12 different glycoprotein species. Two of these glycoproteins (gB and gC) bind heparan sulfate on a cell (Figure 2A and Figure 3(1)). Next, gD binds to entry receptors such as herpes virus entry mediator (HVEM), nectin-1 and 3-O sulfated heparan sulfate. Once bound to the HVEM, gD changes conformation and interacts with viral glycoproteins H (gH) and L (gL), which form a complex. These interactions may result in a hemifusion state. The interaction of gB with the gH/gL complex triggers membrane fusion and creates an entry pore for the delivery of the viral capsid to nuclear pores. Transcription and replication of the viral genome as well as the assembly of progeny capsids take place within the nucleus. The viral mRNA is synthesized by the host cell RNA-polymerase II with the participation of viral factors in all steps in infection. Viral proteins regulate sequential transcriptional cascades (α, β, and γ genes) and a series of posttranslational modifications. After the initiation of viral DNA replication, levels of expression of late γ genes, especially encoding capsid proteins, increase to provide the assembly of progeny virions. Capsid assembly and viral genome packaging occur in the nucleus followed by nucleocapsid egress from the nucleus via nuclear pore or by budding through the nuclear membrane. With the participation of UL36 and UL37 proteins, the capsid is transported from the nucleus to the cytoplasm, where virion maturation and outer shell formation occur. Release of the virion from the cell by exocytosis accomplishes envelope formation.

### 2.2. Hepatitis C Virus (HCV), Zika Virus (ZIKV), West Nile Virus (WNV), Dengue Virus (DENV), and Chikungunya Virus (CHIKV)

HCV, ZIKV, WNV, and DENV are all part of the *Flaviviridae* family and are enveloped, with icosahedral and spherical geometries. HCV, of the genus *Hepacivirus*, is a major cause of liver disease, while ZIKV, WNV, and DENV, of the genus *Flavivirus*, cause a series of prevalent arthropod-borne viral diseases. While HCV and DENV belong to the same family and thus share many features of their life cycles, their virion organization and properties differ substantially. HCV and DENV are enveloped viruses with at diameter of approximately 40–65 nm and they have a non-segmented, single-stranded, 9.6- to 11-kb, positive-sense RNA genome (Figure 2B,C). The core of the viral particle is composed by the capsid proteins, which are thought to enclose the RNA genome (Figure 2B,C). The HCV glycoproteins (envelope 1 [E1] and envelope 2 [E2], Figure 2B), and the DENV glycoproteins (envelope [E] and matrix [M] proteins, Figure 2C) are embedded in the lipid envelope. In case of HCV infection, E1 serves as the fusogenic subunit and E2 acts as the receptor-binding protein. Entry into host cells occurs through complex interactions between virions and several cell-surface molecules (Figure 3(2)). In case of DENV, E and M proteins form the external surface of the mature virus particle. The binding of E protein to dendritic cell specific receptor triggers the internalization of DENV into the cells. Once inside the cells, the genome is translated to proteins, then proteolytically processed by viral and cellular proteases to produce three structural and nonstructural (NS) proteins. The NS proteins recruit the viral genome into an RNA replication complex and the RNA replication takes places via the viral RNA-dependent RNA polymerase. After the negative-strand RNA is synthesized, it serves as a template to produce new positive-strand viral genomes. Nascent genomes can then be translated, further replicated or packaged within new virus particles and released at the cell surface. CHIKV is from the *Togaviridae* family and is also enveloped, with icosahedral and spherical geometry. The diameter is 65–70 nm with a non-segmented, single-stranded, 10- to 12-kb, positive-sense RNA genome. The virus consists of four nonstructural proteins and three structural proteins. The structural proteins are the capsid and two envelope glycoproteins: E1 and E2, which form heterodimeric spikes on the virion surface. E2 binds to cellular receptors to enter the host cell through receptor-mediated endocytosis. E1 contains a fusion peptide which, when exposed to the acidity of the endosome in eukaryotic cells, dissociates from E2 and initiates membrane fusion that allows the release of nucleocapsids into the host cytoplasm, promoting infection.

### 2.3. Influenza A Virus (IAV)

Influenza A virus is part of the *Orthomixoviridae* family, an enveloped, roughly spherical virus with a diameter of about 50–120 nm and eight distinct negative-sense single-stranded RNA genome segments. Influenza virus has three membrane proteins: hemagglutinin (HA), proton pump (M2), and neuraminidase (NA). The inner membrane of the virion is backed by matrix (M1) protein, and the inside of the virion contains eight different genome segments. Each genome segment is a ribonucleoprotein (RNP) complex that consists of a negative-strand RNA genome together with an RNA polymerase complex (PA, PB1, PB), nucleoprotein (NP), and nonstructural proteins (NS) (Figure 2D).

The IAV binds to host cell glycoproteins or glycolipids by HA protein and enters cells through receptor-mediated endocytosis (Figure 3(3)). Under the low pH of the late endosome, HA induces fusion of the viral and endosomal membranes. After the replication and transcription of IAV genomic RNAs takes place in the nucleus by the trimeric viral polymerase complex composed of PB2, PB1, and PA subunits, the viral proteins enter the endoplasmic reticulum. Transport of viral protein to the plasma membrane likely requires host factors. At the plasma membrane, HA and NA associate with lipid rafts that are the site of influenza virus budding. The assembly and virion incorporation of the eight distinct viral ribonucleoproteins requires segment-specific packaging signals in the viral RNAs.

### 2.4. Human Immunodeficiency Virus-1 (HIV-1)

HIV-1 belongs to the *Retroviridae* family and is an enveloped, roughly spherical virus with a diameter of about 120 nm and two copies of a positive-sense single-stranded RNA genome. This single-stranded RNA is bound to integrase, reverse transcriptase, and other proteins. The viral envelope contains proteins from the host cell and relatively few copies of the envelope protein, known as glycoprotein (gp)120, and a stem consisting of three gp41 molecules (Figure 2E).

HIV-1 binds to a CD4 receptor and one of two co-receptors on the surface of a CD4+ T-lymphocyte. The virus then fuses with the host cell (Figure 3(4)). After fusion, the virus releases genomic RNA into the host cell. An HIV enzyme called reverse transcriptase converts the single-stranded HIV RNA to double-stranded HIV DNA. The newly formed HIV DNA enters the nucleus of the host cell, where an HIV enzyme called integrase inserts the HIV DNA within the host cell’s own DNA. The integrated HIV DNA is called a provirus. The provirus may remain inactive for several years, producing few or no new copies of HIV. When the host cell receives a signal to become active, the provirus uses a host enzyme called RNA polymerase to create copies of the HIV genomic material, as well as shorter strands of RNA called messenger RNA (mRNA). The mRNA is used as a blueprint to make long chains of HIV proteins. An HIV enzyme called protease cuts the long chains of HIV proteins into smaller individual proteins. As the smaller HIV proteins come together with copies of the RNA genetic material of HIV, a new virus particle is assembled. The newly assembled virus pushes out from the host cell.

Figure 3 provides representative schematic overviews of the viral life cycles of HSV, HCV, IAV, and HIV-1 in infected cells. In the first step of each virus infection, virus attaches to the cell surface with or without binding receptors. This attachment step is described as “Step A” in this study. Among those viruses, HCV and IAV enter the cell by endocytosis. This entry step is described as “Step B” in this study. In case of HCV and IAV, as the pH in the endosome drops, a conformational change in viral membrane proteins is triggered and induces membrane fusion between virus and cells. This membrane fusion step is described as “Step C” in this study. In the cases of HSV and HIV-1, both Steps B and C are integral steps with Step A. After uncoating viral genes from the virion in the cytoplasm or nucleus, the viral genes are replicated and/or transcribed to mRNA to create progeny virions. This replication step is described as “Step D” in the study. In the last step, newly produced virions are secreted from the infected cell by enzymatic cleavage of the virus-cell interaction. This step is described as “Step E” in this study.

## 3. Antiviral Activity of Catechins on Enveloped DNA Viruses

In 2005, Lyu et al. [5] reported that among flavonoids, flavonols such as EC (**15**) and ECG (**10**) showed strong antiviral activity against HSV-1 (strain KOS, 50% effective concentration (EC_50_) = 2.5 μM and 4 μM, respectively) and HSV-2 (strain G) (EC_50_ = 35 μM and 63 μM, respectively). They also confirmed that pretreatment of Vero cells with **10** before virus adsorption led to slightly enhanced inhibition, as determined by a yield reduction assay (Inhibition step not discussed).

In 2006, Savi et al. [6] reported that EGC (**12**) and GC (**13**), both of which have three hydroxyl groups at the B-ring, caused less DNA damage and the effective concentrations required to inhibit 50% of virus replications (EC_50_) against two different viral strains, HSV-1 (strain KOS) and HSV-1 (strain 29-R), were 173.56 μM and 70.42 μM with **12**, and 103.32 μM and 140.11 μM with **13**, respectively. While C (**16**) and EC (**15**), as compounds with two hydroxyls on the B-ring, against HSV-1 (strain KOS) and HSV-1 (strain 29-R) were less effective and the EC_50_ values were 630.00 μM and 629.38 μM with **16**, and 458.57 μM and 107.14 μM with **15**, respectively. (Inhibition step: D).

In 2008, Isaacs et al. [7] reported that EGCG (**1**) has greater anti-HSV activity than other green tea catechins and inactivates multiple clinical isolates of HSV-1(strain F1) and HSV-2 (strain 333). These viral titers were reduced by more than three orders of magnitude when the virion was exposed to 100 μM of **1**. Incubation of Vero cells with **1** for 48 h prior to infection did not reduce HSV production. Electron microscopy (EM) studies revealed that the virion directly exposed to **1** showed morphological changes and immunogold labeling of envelope glycoproteins gB and gD was significantly reduced by treatment with **1**, whereas capsid protein labeling was unchanged. They confirmed that EGCG produces macromolecular complexes with gB and gD, which are necessary for viral entry in host cells, and inactivates viral infectivity. (Inhibition step: B).

In 2011, Gescher et al. [8] reported that flavan-3-ols such as **10** and **1** and oligomeric proanthocyanidins with galloylation at the 3-OH position such as epicatechin-3-*O*-gallate-(4β→8)-epicatechin-3-*O*-gallate (**21**) inhibited the cytotoxic effect by HSV-1 (strain 17 syn+) infection by approximately 36%, 98%, and 100% at 2 μM, respectively. On the other hand, ungalloylated compounds such as EC (**15**), GC (**13**), and procyanidin B2 (**22**) inhibited only approximately 16%, 3%, and 1% at 2 μM, respectively. Furthermore, **21** caused oligomerization of HSV-1 glycoprotein D (gD). They found that pretreatment of HSV-1 with R2, the aerial parts of *Rumex acetosa* L. containing high amounts of oligomeric and polymeric proanthocyanidins and flavonoids, abolished virus adsorption to the cell surface. These findings corresponded with the report by Isaacs et al. [7] (Inhibition step: A).

In 2011, Isaacs et al. [9] pursued their study on the antiherpetic activity of digallate dimers of EGCG (**1**). From their previous report, 100 μM **1** inactivated HSV-1 (strain F1) and HSV-2 (strain 333) at pH 8.0 by 3 log_10_ to 4 log_10_, but was ineffective at pH 5.7 and 6.6. On the other hand, EGCG digallate dimers such as theasinensin A (**19**), P2 (**20**), and TFDG (**26**) inactivated both viruses at 3 log_10_ to 4 log_10_ at pH 5.7, and by as much as 5 log_10_ at pH 8.0. All EGCG dimers inactivated envelope viruses with class I, II or III (HSV-1, HSV-2) fusion proteins more effectively than did monomeric EGCG. Since HSV-1 glycoprotein B (gB) aggregated more rapidly with theasinensin A than with EGCG, they inferred the EGCG dimer inhibits the function of the viral membrane proteins required for infectivity. (Inhibition steps: A, B).

In 2018, Pradhan et al. [11] reported that 1 min exposure of HSV-1 (strain KOS) to 2.0 μM EGCG (**1**) reduced the virus titer by approximately 40%. Treatment of HSV-1 with 2.0 μM of **1** at 37 °C, room temperature, and 4 °C led to approximately 80%, 98%, and 60% reductions in plaque numbers, respectively. This result indicates that EGCG can inhibit virus adhesion as well as virus entry. (Inhibition steps: A, B).

These reports were summarized in Table 1 and EGCG mainly interfered with the viral membrane protein functions and inhibited the attachment and entry steps of HSV infection. Catechins with 3-galloyl and 5′-OH groups tend to exhibit higher antiviral activity against HSV.

## 4. Antiviral Activity of Catechins on Enveloped (+) ssRNA Viruses

In 2007, Zuo et al. [15] extracted polyphenols from the Chinese medicinal herb *Rhodiola kirilowii* (Rega) Maxim and isolated 12 compounds. These compounds were tested for in-vitro activity against serine protease (NS3-SP) of HCV. As a result, the (−)-epicatechin derivatives: 3,3′-digalloylproprodelphinidin B2 (rhodisin; **18**), epicatechin-3-*O*-gallate-(4β→8)-epicatechin-3-*O*-gallate (**21**), (−)-EGCG (**1**), and ECG (**10**) displayed the most potent activity, with 50% inhibitory concentration (IC_50_) values of 0.77, 0.91, 8.51, and 18.55 μM, respectively. Methylation and acylation of the hydroxyl groups of the above four (−)-epicatechin derivatives reduced this activity. (Inhibition step was not discussed).

In 2011, Ciesek et al. [16] reported that EGCG (**1**) had no effect on HCV RNA replication, assembly, or release of progeny virions. However, **1** inhibited cell-culture-derived HCV (HCVcc) entry into both hepatoma cell lines and primary human hepatocytes. The half-maximal inhibitory concentration of EGCG was approximately 2.5 μg/mL (5.5 μM) and the inhibitory effect was independent of the HCV genotype, and both infection of cells by extracellular virions and cell-to-cell spread were blocked. They summarized that EGCG inhibits HCV entry by blocking viral attachment. (Inhibition steps: A, B).

In 2012, Calland et al. [17] demonstrated that a concentration of 50 μM of EGCG (**1**) inhibited HCV infectivity by more than 90% at an early stage of the virus cycle, most likely the entry step. They reported that **1** prevents virus attachment to the cell surface, probably by acting directly on the virion. However, **1** showed no effects on viral replication or virion secretion. In addition, **1** inhibited HCV cell-to-cell spread. This inhibition was not observed in members of the *Flaviviridae* family such as bovine viral diarrhea virus (BVDV), yellow fever virus (YFV), or sindbis virus (SINV). (Inhibition steps: A, B).

In 2015, Calland et al. [19] continuously studied the antiviral activity of EGCG (**1**) against HCV and the mechanisms of action involved. Cryo-transmission EM observations of HCV pseudoparticles treated with EGCG identified a bulge on those particles. They concluded that EGCG inhibits HCV entry by altering the particle structure and impairing attachment to the cell surface. (Inhibition step: A).

In 2016, Carneiro et al. [20] reported that treatment of ZIKV^BR^ (Clinical strain) or ZIKV MR766 (African strain) with EGCG (**1**) inhibited their focus forming activities by at least 1-log (>90%) at 100 μM and interfered with virus entry into Vero E6 cells. However, pre-treatment of cells with EGCG did not show any effects on virus attachment. (Inhibition steps: A, B).

In 2017, Sharma et al. [22] studied the mechanisms of action by which EGCG (**1**) inhibited ZIKV infection using molecular docking simulations. EGCG was found to interact with several residues of the E protein and interfered with the membrane fusion step by blocking the fold-back event. (Inhibition step: C, but only by docking simulation).

That the same year, Vázquz-Calvo et al. [23] assayed the effect of a series of polyphenols on WNV-NY99 infection and found that delphinidin (**14**) and EGCG (**1**) reduced the viral titer by more than three orders of magnitude as the virus pretreated with 10 μM of each polyphenol. These compounds mainly affected the attachment and entry steps of the virus life cycle. This antiviral activity against WNV is produced by virucidal effects rather than by inhibition of pH-dependent viral fusion. Both polyphenols also reduced the infectivity of ZIKV MR766, ZIKV PA259459, and DENV-2. (Inhibition steps: A, B).

In 2017, Lu et al. [25] confirmed that approximately 40 μM of EGCG (**1**) inhibited three different CHIKV (S27 ATCC-VR-64, Singapore/0611aTw/2006/FJ807896, Malaysia/0810bTw/2008/FJ807899). They found that **1** inhibited the entry, replication and release of CHIKV in vitro and co-treatment with suramin further enhanced anti-CHIKV effects. (Inhibition steps: B, D, E).

In 2018, Raekiansyah et al. [24] reported that EGCG (**1**) inhibited DENV infection regardless of the infecting serotype and the 50% effective concentration of EGCG in Vero cells was estimated to be approximately 14.8 μM, 18.0 μM, 11.2 μM, and 13.6 μM for DENV-1, DENV-2, DENV-3, and DENV-4, respectively. However, no or minimal inhibition was observed with other flavivirus, including JEV, YFV, and ZIKV, contradicting previous reports by Carneiro [20] and Vázquz-Calvo [23]. In those reports, ZIKV particles were incubated with EGCG for 1 h before the mixture was applied to cells. Virus yield was determined by focus assay at 96 h post-infection or by plaque assay at 24 h post-infection. The experimental conditions used by Raekiansyah et al. exposed cells directly to virus particles together with EGCG, with incubation for 5 days before the virus antigen level present in infected culture fluid was measured by enzyme-linked immunosorbent assay (ELISA). Such differences in procedures could have led to the discrepancies in results between their study and previous investigations. (Inhibition steps: A, B).

From these reports were summarized in Table 2 and EGCG most likely inhibits the attachment and entry steps used by HCV, ZIKV, DENV, and WNV, but not those of BVDV, JEV and YFV. This is probably due to the structural differences in these viral proteins, with EGCG thus acting differently to each virus.

## 5. Antiviral Activity of Catechins on Enveloped (−) RNA Virus

### 5.1. Antiviral Mechanism of Action of EGCG on HIV-1

In 1994, Chang et al. [27] investigated the HIV reverse transcriptase inhibitory effects of EGC (**12**), ECG (**10**), and EGCG (**1**), and found that the IC_50_ values of each compound were 7.80, 0.32, and 0.68 μM, respectively. Both **10** and **1**, with a galloyl group at the 3-position, are more potent inhibitors than **12**. A kinetic study revealed that polyphenolic catechins bind to the template primer-binding site in reverse transcriptase and inhibit enzyme activity. (Inhibition step: D, but only against purified enzyme).

In 2001, Tillekeratne et al. [28] reported that ECG (**10**) and EGCG (**1**), which have galloyl groups at the 3-position, inhibited wild-type HIV-1 reverse transcriptase at sub-micro molar concentrations (IC_50_ = 0.76 μM and 0.73 μM), but EC (**15)** without a galloyl group at the 3-position did not inhibit the reverse transcriptase activity even at 100 μM concentration. (Inhibition step: D, only examined against polymerase).

In 2003, Kawai et al. [29] analyzed the effects of EGCG (**1**) on antibody binding and binding of gp120, an envelope protein of HIV-1, to CD4 using sandwich ELISA and flow cytometry, respectively. Compound **1** binds to CD4 and interferes with gp120 binding to CD4 approximately 70% at 100 μM. These results suggest a preventive effect of EGCG on HIV-1 infection by interfering with CD4 binding activity to gp120. (Inhibition step: A, but not whole virion).

In 2005, Liu et al. [30] reported that catechins that possess a galloyl group at the 3-position and a hydroxyl group at the 5′-position such as (−)-GCG (**3**), EGCG (**1**), epigallocatechin 3,5-digallate (EGCDG; **5**) and theaflavin derivatives inhibited HIV-1 infection by interfering p24 production, cell-cell fusion, and virus-cell fusion at 2.63−9.89 μM, 7.55–12.86 μM, and 2.41–3.44 μM respectively. On the other hand, catechin does not possess a hydroxyl group at the 5′-position such as ECG (**10**), which did not inhibit HIV-1 infection. **3**, **1**, and **5** also inhibited HIV-1 entry into target cells by interfering with the gp41 six-helix bundle formation. (Inhibition step: B, C, and D).

In 2006, Williamson et al. [31] described the binding of EGCG (**1**) to CD4 by nuclear magnetic resonance (NMR) spectroscopy. The addition of CD4 to **1** induced a linear decrease in signal intensity. The binding between **1** and CD4 was strong (*K*_d_ = 10 nmol/L) enough to reduce gp120-CD4 binding in saturation transfer difference (STD) experiments. Under physiologically relevant level of EGCG in plasma (0.2 μM), they observed 40% of HIV-1-gp120 binding to CD4^+^ T cells. Molecular modeling studies described **1** binding to CD4 at the D1 domain, as the gp120-binding pocket. These results suggested that **1** binds to CD4 on the cell, inhibiting interactions between CD4 and gp120 on HIV and exerting anti-virus activities. (Inhibition step: A, only examined against CD4).

In 2009, Nance et al. [32] reported that EGCG (**1**) provided dose-dependent inhibition of HIV-1 p24 antigen production in CD4^+^ T cells. On the other hand, the absence of an effect from EGC (**12**) (lacking the galloyl moiety), ECG (**10**) (lacking the pyrogallol moiety) and (−)-catechin (**16**) (lacking both pyrogallol and galloyl moieties) demonstrated the specificity of the EGCG-induced effect, which requires both pyrogallol and galloyl moieties. **1** inhibited a broad range of HIV-1 subtypes (B, C, D, and G) and receptors used (R5, X4, and X4/R5) at physiological concentrations (IC_50_ = 4.5–12.0 μmol/L) without decreasing cell viability. (Inhibition step not discussed).

In 2010, Jiang et al. [33] showed the HIV-1 integrase inhibition activity of four catechins with the galloyl group, CG (**11**), EGCG (**1**), (−)-GCG (**3**), and ECG (**10**) using ELISA. In addition, the docking study proposed two observations: when viral DNAs do not combine with the integrase, catechins may bind to Try143 and Gln148, thereby altering the flexibility of the loop (140–149), which could inhibit integrase activity; and when viral DNAs combine with integrase, catechins may bind between the integrase and viral DNAs, consequently disrupting integration (IC_50_ = 0.1 μM). (Inhibition step: D, but only examined against integrase and in silico study).

In 2011, Li et al. [34] investigated the mechanism of inhibition on HIV-1 by EGCG (**1**) using HeLa-CD4-LTR-β-gal cells with HIV-1_IIB_ and HIV-2_EHO_. The activity of **1** for blocking HIV-1_IIIB_ and HIV-2_EHO_ replication by 50% (EC_50_) was determined with the multinuclear activation of galactosidase inhibitor and identified as 1.6 and 2.0 μM, respectively. Using a time of addition assay, the profile of the anti-virus activity of **1** was compared with that of other representative HIV-1 inhibitors, including an entry inhibitor (DS5000), a CXCR4 antagonist (AMD3100), a nucleoside RT inhibitor (AZT), and a non-nucleoside RT inhibitor (MKC-442). The profile of **1** was identical to that of MKC-442, thereby suggesting that EGCG inhibits HIV-1 RT activity as an allosteric inhibitor. (Inhibition step: D)

In 2012, Hartjen et al. [35] considers that enhancement of HIV-1 infectivity is mediated by amyloid filaments from peptides that are proteolytically released from prostatic acid phosphate (PAP248–286), termed semen-derived enhancer of virus infection (SEVI). They visualized fibrils in human semen that resembled fibrils formed from synthetic PAP248–286. They demonstrate that the semen-mediated enhancement of HIV-1 infectivity is highly variable and that EGCG (**1**) can abrogate this activity at 0.4 mM, which is a non-toxic concentration, in most semen samples. (Inhibition step: Other).

In 2015, Castellano et al. [36] investigated the anti-SEVI activity of EGCG (**1**) in greater detail. They confirmed that **1** can remodel all four classes of seminal amyloid, PAP248–286, PAP85–120, SEM1(45–107), and SEM2(49–107) at 200 μM and the viral infectivity was reduced to ~61%, ~35%, and 11% at 0.25 μM against the HIV-1 strain BL2, Bal, and 89.6, respectively. (Inhibition step: Other).

From these reports, summarized in Table 3, EGCG (**1**) was found to inhibit multiple steps of the infection process, not only in the early stage, but also in the late stage of HIV-1 infection. Methods for enhancing the binding affinity of EGCG to the virus surface and effective transfection methods are thus requested for further development of anti-HIV drugs. Catechins with 3-galloyl tend to exhibit higher antiviral activity against HIV-1.

### 5.2. Antiviral Mechanism of EGCG on Influenza Virus

In 1949, Green [38] first demonstrated the antiviral activity of green tea extract as the inhibition of influenza virus propagation within chicken embryonated eggs. (Inhibition step: not discussed)

In 1993, Nakayama et al. [2] focused on the major catechin components of tea extract, EGCG (**1**) and TFDG (**26**), and reported that infection of Madin-Darby Canine Kidney (MDCK) cultured cells were inhibited as each 1- to 10-μM solution was allowed to act in advance with influenza A/H1N1 virus and influenza B virus inoculation. On the other hand, these compounds did not show any marked inhibitory effect against virus on MDCK cells already infected with influenza viruses. **1** and **26** also inhibited both hemagglutination of influenza virus with erythrocytes and adsorption of the virus into MDCK cells. These results indicate that **1** and **26** interact with hemagglutinin on the viral membrane and interfere with virus infection. (Inhibition step: A)

In 2002, Imanishi et al. [39] reported that pretreatment of MDCK with green tea extracts prevented acidification of the endosome in MDCK cells and influenza A/H1N1, A/H3N2, and B were inhibited by three orders of magnitude. (Inhibition step: C)

In 2005, Song et al. [40] reported that EGCG (**1**) and ECG (**10**), both of which have a galloyl group at the 3-position of catechin, inhibited infectivity to MDCK cells, hemagglutination activity with erythrocytes, RNA synthesis by viral RNA polymerase, and neuraminidase activity of influenza A/H1N1, A/H3N2, and B viruses. However, EGC (**12**) did not exhibit marked inhibitory effects on viruses. This result suggests that a galloyl group at the 3-position is crucial to the viral inhibitory effect and a hydroxyl group at the 5′-position in the B-ring does not affect antiviral activity. (Inhibition steps: A, D, and E)

In 2007, Furuta et al. [41] evaluated anti-virus activity by inoculating virus into MDCK cells after pre-incubating virus with EGCG (**1**) or dideoxy-epigallocatechin gallate (DO-EGCG; **4**). Anti-virus activity of DO-EGCG was three-fold higher than that of **1** (**4:** IC_50_ = 11.92 μM; **1:** IC_50_ = 41.25 μM), indicating that the hydroxyl groups on the A-ring are not essential for anti-virus activity. (Inhibition step: Step A)

In 2009, Kuzuhara et al. [42] reported that EGCG (**1**) interferes with the endonuclease activity of influenza virus RNA polymerase. According to the structure and the anti-influenza virus activity of catechin derivatives, they proposed that the 3-position galloyl group of **1** and ECG (**10**) enhances the stability of catechin binding to the active catalytic site of endonuclease. (Inhibition step: Step D, but not used on whole virus)

In 2012, Zu et al. [43] reported that TF (**23**) and its derivatives, TF-3-G (**24**), TF-3′-G (**25**), and TFDG (**26**), inhibited hemagglutinin and neuraminidase of influenza A/PR/8/34(H1N1), A/Sydney/5/97(H3N2), and B/Jiangsu/10/2003 viruses at 10–50 μg/mL. Interestingly, **23**, **24**, **25**, and **26** also suppressed the expression of interleukin-6, one of the inflammatory cytokines that induces tissue damage and cell apoptosis. (Inhibition step: Other)

In 2012, Ling et al. [44] suggested that the anti-influenza A/Yamagata/120/86(H1N1) virus activity of EGCG (**1**) is dose- and time-dependent in vitro. The effect of **1** increased significantly when **1** was added 0–6 h after virus inoculation to MDCK cells, whereas the effect was not seen even with pre-treatment of MDCK cells with **1** for 2 h. Among in vivo virus-infected mice, oral administration of 40 mg·kg^−1^·d^−1^ of **1** in BALB/c mice markedly improved the survival rate from 16.7% to 66.7% and its activity was comparable to that of oseltamivir, a major anti-influenza drug. These results showed that the main anti-virus activity of **1** appeared in the early steps of viral replication after attachment of virus to the host cell. (Inhibition step: B)

In 2012, Kowalinski et al. [45] described the high-resolution X-ray cocrystal structure of the 2009 pandemic H1N1 (pH1N1) PA endonuclease domain with the inhibitor including EGCG (**1**). (Inhibition step: D, not used on whole virus)

In 2013, Kim et al. [46] also described EGCG (**1**) directly affecting a series of influenza A/H1N1, A/H3N2, and B virus particles at 5.8–17.3 μM. **1** reduced viral membrane integrity in morphological and physical analyses, decreasing cell invasion efficiency of the virus. In addition to **1**, dimeric EGCG (bEGCdG; **17**) was evaluated using a cytopathic effect reduction assay. Compound **1** showed higher anti-virus activity than **17** for many virus subtypes. They also referred to the partial NA inhibitory effect of **1** using an NA fluorescent substrate. Compound **1** had IC_50_ values of >500 μM against influenza A/PR/8/34(H1N1) and 233.7 μM against NA(H275Y), much higher than the concentration required to induce virus inhibition. These results suggest that the NA inhibitory effect of **1** could contribute partially to the suppression of virus entry (Inhibition steps: A, E)

In 2014, Colpitts et al. [10] showed that EGCG (**1**) inhibited a series of influenza A viruses (PR/8/34(H1N1), USSR/90/77(H1N1), Aichi/2/1968(H3N2), and PC/1/73(H3N2)) at low concentration (EC_50_ = 7.3 to 40.1 μM). After pre-exposing octadecyl rhodamine B chloride-labeled influenza A virus (IAV) to EGCG, the IAV was adsorbed onto MDCK cells at 4 °C to avoid fusion. As a result, the attachments of the IAV to MDCK cells were inhibited by EGCG at 22.9 μM. Additional hemagglutination inhibition assays showed that EGCG inhibited hemagglutination more effectively than monomeric sialic acid. This result indicates that EGCG competes with sialic acid for binding to influenza A virus. (Inhibition step: A)

In 2015, Müller et al. [47] evaluated the interaction between EGCG (**1**) and influenza virus neuraminidase (NA) using a molecular-based matrix assisted laser desorption/ionization (MALDI) mass spectrometry. MALDI mass spectra of treated NA decreased at EGCG binding sites and showed the interaction of NA with EGCG. Computational molecular docking found that EGCG binds to NA at a cavity adjacent to a secondary sialic acid-binding site. The IC_50_ of EGCG in the neuraminidase inhibition assay was 100 μM. (Inhibition step: E, not whole virion)

In 2017, Quosdorf et al. [48] analyzed the relationship between structure and anti-activity for flavan-3-ols including EGCG (**1**) by 2′-(4-methylumbelliferyl)-α-d-*N*-acetylneuraminic acid (MUNANA)-based activity assay. Compared to oseltamivir carboxylate and zanamivir, anti-NA activities were weak with all flavan-3-ols. However, (+)-GCG (**2**) exhibited the highest activity of all flavan-3-ols. This relationship showed that the presence of galloyl groups and the hydroxylation pattern of the flavan skeleton are essential for anti-neuraminidase activity. (Inhibition step: E, not whole virion)

From these reports were summarized in Table 4 and EGCG (**1**) was found to most likely interact with hemagglutinin and inhibit virus attachment. The virus inhibitory effect was drastically increased when the virion was pre-treated with **1** before inoculation. Compound **1** and catechins with 3-galloyl group such as TF-3-G, TF-3′-G, TFDG, and (+)-GCG directly inhibited neuraminidase activity and virus infection.

## 6. Structure and Antiviral Activity of Catechin Derivatives

Based on previous reports summarized in this review, the structure and antiviral activity relationships of catechin derivatives are summarized in Table 5. Although the target molecules differ, catechins with 3-galloyl and 5′-OH groups tend to exhibit higher antiviral activity against HCV, HIV-1, and influenza A virus than other catechins.

## 7. Lipid Bilayer Affinity of EGCG-Alkyl Ether Derivatives

In 1998, Kouno et al. [49] reported that EGCG (1) inhibited lipid peroxidation in liposome bilayer caused by water soluble radical initiator [2,2′-azobis(2-amidinopropane) dihydrocholoride (AAPH)]. But 1 did not inhibit peroxidation caused by lipophilic radical initiator [2,2′-azobis (2,4-dimethylvaleronitrile) (AMVN)], because it does not penetrate into the hydrophobic region of lipid bilayer. Therefore, they synthesized a series of lipophilic EGCG such as thioether derivatives (6; EGCG-(CH_2_-S-C8)_2_ and EGCG-(CH_2_-S-Bn)_2_) and a *n*-octadecylisocyanate derivative (7; EGCG-C18-Carbamoyl). These compounds were evaluated their inhibition activities against lipid peroxidation of egg-phosphatidylcholine (PC) by measuring the concentration of thiobarbitulic acid reactive substrate. As a result, EGCG-(CH_2_-S-C8)_2_ and EGCG-C18-Carbamoyl inhibited lipid peroxidation in liposome lipid bilayer caused both by AAPH at 7.9 μM and 24.6 μM and by AMVN at 60.0 μM and 22.1 μM, respectively (Table 1). EGCG-(CH_2_-S-Bn)_2_ efficiently inhibited lipid peroxidation by AAPH (IC_50_ = 9.6 μM), but showed limited inhibitory effect to AMVN (IC_50_ = 432 μM). These data indicated that EGCG modified with a long alkyl chain obtain increased affinity into the hydrophobic region of the lipid bilayer.

## 8. EGCG-Fatty Acid Derivatives

### 8.1. Synthesis of EGCG-Fatty Acid Derivatives

Since EGCG modified with a long alkyl chain possesses lipophilic property [49], we synthesized a series of EGCG fatty acid monoester derivatives **8** by lipase-catalyzed transesterification [50]. EGCG-monoesters modified with saturated fatty acids such as butanoyl, octanoyl, lauroyl, palmitoyl, and stealoyl groups are represented as EGCG-C4, EGCG-C8, EGCG-C12, EGCG-C16, and EGCG-C18, respectively. EGCG-monoesters modified with non-saturated fatty acids such as linoleyl and linoneyl are represented as EGCG-C18DE and EGCG-C18TE.

Although EGCG (**1**) has been reported to interfere with the catalytic activity of lipases [51], we succeeded in preparing EGCG-fatty acid derivatives by a lipase-catalyzed transesterification in polar organic solvents such as *N*,*N*-dimethylformamide or acetonitrile [50]. Interestingly, this lipase-catalyzed method afforded the B-ring modified esters as major products in case the acyl donor is a saturated fatty acid ester such as vinyl stearate (Acyl position, R^1^:R^2^:R^3^:R^4^ = 38:35:7:20) [50], while it affords the D-ring modified esters as major products in case the acyl donors are non-saturated fatty acid such as vinyl linolate (Acyl position, R^1^:R^2^:R^3^:R^4^ = 28:22:5:45) and linoleate (Acyl position, R^1^:R^2^:R^3^: R^4^ = 15:19:4:62) according to ^1^H-NMR spectroscopy analysis.

### 8.2. Cytotoxicity and Influenza Virus Inhibitory Effect of EGCG and EGCG-Fatty Acid Derivatives

In 2008, Mori et al. [50] examined cytotoxicity of EGCG (**1**) and EGCG derivatives (**8**) to MDCK cells by MTT proliferation and viability assay. As a result, the cytotoxicity of EGCG-fatty acid derivatives (**8**) was increased in an alkyl-length dependent manner. Interestingly, the lauroyl ester showed the highest among them and the CC_50_ was 42 μM which was 6.6-fold higher than **1** (CC_50_ = 275 μM) [50]. This was probable due to the increased cellular membrane affinity of EGCG-C12 with modest water solubility.

In this review, we further provide the effect of non-saturated bonds in EGCG-fatty acid esters on the cytotoxicity by synthesizing EGCG-fatty acid esters that have the same alkyl chain length but have different number of *cis*-olefin bonds. As a result, EGCG-C18TE possesses three *cis*-olefin bonds in C18 alkyl chain showed 9.4-fold higher cytotoxicity (CC_50_ = 32 μM) than EGCG-C18 (CC_50_ = 300 μM) (Table 6). On the other hand, EGCG-C18DE that has two *cis*-olefin bonds in C18 alkyl chain showed only 1.2-fold higher cytotoxicity (CC_50_ = 250 μM) than EGCG-C18 (Table 6). The hydrophobicity of EGCG-C18, EGCG-C18DE, and EGCG-C18TE were estimated as log P value using the ChemBioDraw Ultra ver. 11 software and those log P values were identified as 8.97, 8.33, and 8.01, respectively. Thus, the higher cytotoxicity of EGCG-C18TE could be due to both better water solubility and the higher cellular membrane affinity.

Anti-influenza virus activities of **1** and EGCG-fatty acid derivatives (**8**) were studied using two different experimental methods. In the first experiment, **1** and EGCG fatty acid derivatives were added to a confluent monolayer of MDCK cells, followed by incubation for 2 h at 37 °C. After the solution was removed from each well, cell sheets were washed and infected with influenza A/PR/8/34 (H1N1) virus. After 1 h for virus adsorption at room temperature, the cell sheets were washed and assessed by the plaque formation inhibitory assay. As a result, the EC_50_ of EGCG-C18DE and EGCG-C18TE were 7.0 μM and 3.0 μM that were much lower than **1** (EC_50_ = 94 μM) and EGCG-C18 (EC_50_ = 64 μM) (Table 6). The selectivity index (SI) of EGCG-C18DE was 35.7 that is higher than **1** (SI = 2.91) and EGCG-C18 (SI = 4.68) (Table 6).

In the second experiment, the virus was pre-treated with **1** or EGCG-fatty acid derivatives (**8**) to assess their virucidal activities. Briefly, each compound was directly mixed with influenza A/PR/8/34(H1N1) virus and incubated for 30 min at room temperature. The mixed solution was then applied to a confluent monolayer of MDCK cells and subsequently assessed by plaque formation inhibitory assay. As a result, EGCG-C18 exhibited higher virucidal activity (EC_50_ = 60 nM in Table 7) than EGCG-C18DE (EC_50_ = 180 nM) and EGCG-C18TE (EC_50_ = 100 nM) and those were less concentration used in Table 6. From the SI values of **1** and EGCG-fatty acid derivatives summarized in Table 6 and Table 7, these compounds directly interact with the virus particle rather than cells and show potent virucidal activity at much lower concentrations. Further, the introduction of fatty acids such as stearoyl, linoleyl, and linolenyl derivatives to EGCG drastically enhance the antiviral activity of **1**. The virucidal effect of EGCG-fatty acid derivatives was also confirmed for other seasonal influenza A/H1N1, A/H3N2, B viruses and avian influenza A/H5N2 viruses [52]. From these data, EGCG-fatty acid esters considered to interact with some common regions of virus components such as viral membrane or proteins and interfere the attachment, entry and membrane fusion (Inhibition Step: A–C).

### 8.3. Anti-Influenza Virus Activity of EGCG-C16 in Chicken Embryonated Eggs

In 2009, Kaihatsu et al. [52] further assessed the virucidal effect of EGCG-fatty acid derivatives on avian influenza virus in chicken embryonated eggs. As this effect has already been assessed in cell-based assays, they pre-treated with influenza A/Duck/Hong Kong/342/78 (H5N2) to EGCG (**1**), EGCG-C16 (**8**), zanamivir, and oseltamivir phosphate at a concentration of 1 μM for 1 h at room temperature. The mixture was then inoculated (50 pfu/egg) into the allantoic fluid of embryonated eggs for 7 days at 37 °C. As a result, EGCG-C16 completely inhibited avian influenza virus infection of chicken embryos, while commercially available drugs did not show complete blockage [53,54]. These results indicate that EGCG-C16 induces irreversible and virucidal denaturing of influenza viruses.

### 8.4. Antiviral Activity of EGCG-Fatty Acid Derivatives for Other Viruses

In 2012, Zhong et al. [55] prepared lipophilic ester derivatives of EGCG, namely EGCG-*O*-tetrastearate, EGCG-*O*-tetraeicosapentaenoate, EGCG-*O*-tetradocosahexaenoate, and EGCG-*O*-octabutylate (**9**). The EGCG-polyunsaturated fatty acids showed approximately 1700-fold and 22-fold higher anti-hepatitis C virus protease inhibitory activities than the positive control embelin and EGCG-*O*-tertrastearate in vitro. (Inhibition step; D, but only tested against protease)

In 2013, Oliveira et al. [56] reported that palmitoyl-EGCG (**8**, EGCG-C16) blocks viral glycoprotein(s) and efficiently inhibit the binding of HSV-1 to host receptors. (Inhibition step: A)

In 2014, Zhao et al. [26] evaluated the antiviral activity of EGCG-*O*-monopalmitate (**8**, EGCG-C16) against Porcine Reproductive and Respiratory Syndrome Virus (PRRSV). EGCG-C16 showed 177-fold higher the virus inhibitory effects compared to EGCG when they were added to a monolayer of MARC-145 cells prior to the virus inoculation at 10 TCID_50_ (50% tissue culture infectious dose). They infer that EGCG-C16 may inhibit viral adsorption and cell intrusion. The EC_50_ value of EGCG-C16 for PRRSV is nearly the same level as Ribavirin, a guanosine analog used to inhibit viral RNA synthesis, but the selectivity is 3.8-fold higher than them. (Inhibition step: A). From these reports, EGCG-fatty acid esters efficiently interact with the viral membrane or the cellular membrane and prevent viral attachment and entry steps.

## 9. Conclusions

From this study, EGCG was found to be the most potent and universal virus inhibitor among the natural catechins, directly interacting not only with various types of enveloped DNA, (+)-RNA, and (−)-RNA viruses, but also various types of cells. The 3-galloyl and 5′-OH groups appear crucial for virus inhibition activity. EGCG mainly inhibits the early stages of infections, such as attachment, entry, and membrane fusion, by interfering with either viral membrane proteins or cellular protein or both of them. We thus developed EGCG-fatty acid derivatives to improve the viral and cellular membrane permeability of EGCG and investigated their antiviral activities. As a result, EGCG-fatty acid monoesters with a long fatty acid showed improved antiviral activities against broad spectrum of influenza virus, HSV, and PRRV, with higher potency than EGCG. This methodology may facilitate the application of EGCG-fatty acid derivatives to the prevention and treatment of viral infections.

## Figures and Tables

**Table 1:** Summary of Antiviral Activity of Catechins on Enveloped DNA viruses.

Compound	Virus	Assay	Activity	Ref.
ECG	HSV-1 F1/HSV-2 333	Cytopathic effect inhibition	EC_50_ = 4.0/63.0 μM	[5]
EC			EC_50_ = 2.5/35.0 μM	
EGC	HSV-1 KOS/HSV-1 29R	Virus replication inhibition	EC_50_ = 173.6/70.4 μM	[6]
GC			EC_50_ = 103.3/140.1 μM	
C			EC_50_ = 630.0/629.4 μM	
EC			EC_50_ = 458.6/107.1 μM	
EGCG	HSV-1 F1/HSV-2 333	Titer reduction	10^2.0^/10^4.4^ reduction at 100 μM	[7,9]
Theasinensin A			10^4.0–5.0^/10^4.0^ reduction at 100 μM	
EGCG	HSV-1 17 syn+	Cytotoxicity inhibition	98% at 2.0 μM	[8]
ECG			36% at 2.0 μM	
EC			16% at 2.0 μM	
GC			3% at 2.0 μM	
EGCG	HSV-1 KOS	Plaque formation inhibition	60% inhibition (2.0 μM at 4 °C) 98% inhibition (2.0 μM at RT) 80% inhibition (2.0 μM at 37 °C)	[11]

EC_50_: The EC_50_ represents the concentration of compound required to reduce virus infectivity by 50% relative to the control well without test compound. RT: room temperature.

**Table 2:** Summary of Antiviral Activity of Catechins on Enveloped (+) ssRNA Viruses.

Compound	Target	Assay	Activity	Ref.
EGCG	HCV serine protease (NS3-SP)	Serine protease inhibition	IC_50_ = 8.51 μM	[15]
ECG			IC_50_ = 18.55 μM	
EGCG	HCVcc	Luciferase reporter	IC_50_ = 5.5 μM	[16]
EGCG	HCVcc	Luciferase reporter	IC_50_ = 5.0 μM	[17]
EGCG	ZIKV ^BR^/ZIKV MR766	Focus forming inhibition	>90% inhibition at 100 μM	[20]
EGCG	WNV-NY99	Plaque forming inhibition	>10^4.0^ reduction at 10 μM	[23]
	ZIKV MR766		>10^4.0^ at 10 μM	
	ZIKV PA259459		>10^3.0^ at 10 μM	
	DENV-2		>10^3.0^ at 10 μM	
EGCG	CHIKV S27	Microneutralization	IC_50_ = 1.99 μg/mL (4.34 μM)	[25]
EGCG	DENV-1	Antigen reduction	EC_50_ = 14.8 μM	[24]
	DENV-2		EC_50_ = 18.0 μM	
	DENV-3		EC_50_ = 11.2 μM	
	DENV-4		EC_50_ = 13.6 μM	

The IC_50_ represents the concentration of compound required to inhibit virus infectivity by 50% relative to the control well without test compound.

**Table 3:** Summary of Antiviral Activity of Catechins on HIV-1.

Compound	Target	Assay	Activity	Ref.
EGCG	HIV-1 purified reverse transcriptase	Reverse transcriptase inhibition	IC_50_ = 0.68 μM	[27]
ECG			IC_50_ = 0.32 μM	
EGC			IC_50_ = 7.80 μM	
EGCG	HIV-1 purified reverse transcriptase	Reverse transcriptase inhibition	IC_50_ = 0.73 μM	[28]
ECG			IC_50_ = 0.76 μM	
EGCG	HIV-1 CD4 protein	Anti-CD4 binding to CD4 protein inhibition	Approx. 70% inhibition at 100 μM	[29]
EGCG/GCG	HIV-1_IIIB_	p24 antigen production	IC_50_ = 9.89 μM/4.61 μM	[30]
	HIV-1_IIIB_	Cell-cell fusion	IC_50_ = no inhibition/7.55 μM	
	HIV-1_NL4-3_-*luc* pseudotyped with HIV-1_HXB2_	Virus-cell fusion	IC_50_ = 3.44 μM/2.45 μM	
EGCG	HIV-1 gp120	Inhibition of gp120 binding to CD4^+^ cells	40% inhibition at 0.2 μM	[31]
EGCG	HIV-1_SF162_ (R5)	p24 antigen production	IC_50_ = 4.5 μM	[32]
	HIV-1_89.6_ (X4/R5)		IC_50_ = 8.0 μM	
	HIV-1_92UG038_ (X4)		IC_50_ = 9.0 μM	
	HIV-1_JV1083_ (R5)		IC_50_ = 9.0 μM	
EGCG/EGC	HIV-1 _IIIB_	Multinuclear activation of galactosidase inhibition	EC_50_ = 1.6 μM/3.4 μM	[34]
	HIV-2 _EHO_		EC_50_ = 2.0 μM/7.9 μM	
EGCG	HIV-1_BAL_(X5)	Semen-derived enhancer of virus infection monitored by luciferase reporter expression	Inhibited > 70.6% at 0.4 mM	[35]
	HIV-1_NL4/3_ (X4)			
	HIV-1B (isolate)			
EGCG	HIV-1_BL2_	Semen-derived enhancer of virus infection monitored by luciferase reporter expression	~61% inhibition at 0.25 μM	[36]
	HIV-1_BAL_		~35% inhibition at 0.25 μM	
	HIV-_89.6_		~11% inhibition at 0.25 μM	

**Table 4:** Summary of Antiviral Activity of Catechins on influenza virus.

Compound	Target	Assay	Activity	Ref.
EGCG/ TFDG	A/Yamagata/120/86(H1N1)	Plaque forming inhibition	100% inhibition at 1.5 μM/ 100% inhibition at 1.5 μM	[2]
	B/USSR/100/83		100% inhibition at 1.5 μM/ 100% inhibition at 1.5 μM	
EGCG/ ECG/EGC	A/Chile/1/83(H1N1)	Plaque forming inhibition	EC_50_ = 28.4/26.4/318 μM	[40]
	A/Sydney/5/97(H3N2)		EC_50_ = 22.8/22.2/309 μM	
	B/Yamagata/16/88		EC_50_ = 26.1/40.4/311.1 μM	
EGCG/ DO-EGCG	A/Memphis/1/71(H3N2)	Focus forming inhibition	IC_50_ = 41.25/11.92 μM	[41]
EGCG/ ECG	N-terminal endonuclease domain protein of A/PR/8/34(H1N1) RNA polymerase PA	Endonuclease inhibition	100% inhibition at 10 μM/ 100% inhibition at 10 μM	[42]
TF, TF-3-G/ TF-3′-G/ TFDG	A/PR/8/34(H1N1)	NA inhibition	IC_50_ = 11.65 μg/mL (TF), IC_50_ = 31.91/35.23/26.25 μM	[43]
	A/Sydney/5/97(H3N2)		IC_50_ = 25.72 μg/mL (TF), IC_50_ = 13.29/18.26/10.67 μM	
	B/Jiangsu/10/2003		IC_50_ = 27.98 μg/mL (TF), IC_50_ = 49.60 /49.23 /42.07 μM	
EGCG	A/Yamagata/120/86(H1N1)	Lethal murine infection model	Survival rate improved from 16.7% to 66.7% at 40 mg·kg^−1^·d^−1^ oral administration	[44]
EGCG	A/Puerto Rico/8/34 (H1N1)	NA activity inhibition	IC_50_ > 500 μM	[46]
	Purified NA from A/California/04/2009 (H275Y)		IC_50_ = 233.7 μM	
EGCG	A/PR/8/34(H1N1), A/USSR/90/77 (H1N1), A/Port Chalmers/1/73(H3N2), A/Aichi/2/68(H3N2)	Plaque forming inhibition	EC_50_ = 7.3~40.1 μM	[10]
EGCG	A/California/04/2009(H1N1)	NA inhibition	IC_50_ =1565 μM	[48]
(+)-GCG			IC_50_ = 396 μM	

**Table 5:** Comparison of Antivirus Activity of Natural Catechins.

Target Virus	Virus Inhibitory Effect	Important Functional Group	Targets	Ref.
HSV	EGC > EC, GC > C	5′-OH	Entire infection process	[6]
HCV	EGCG > ECG	5′-OH	NS3-Serine Protease	[15]
HIV-1	ECG, EGCG > EGC	3-galloyl	Reverse transcriptase	[27]
HIV-1	EGCG > EGC > ECG > C	3-galloyl, 5′-OH	Glycoprotein(gp120)	[32]
Influenza	EGCG > ECG > EGC	3-galloyl	Hemagglutinin, viral RNA synthesis, Neuraminidase (NA)	[40]
Influenza	EGCG > ECG > bEGCdG > EGC	3-galloyl, 5′-OH	Viral envelope, NA surface glycoprotein	[46]

**Table 6:** Cytotoxicity and Protective Effect of EGCG and EGCG-Fatty Acid Derivatives against Influenza A/PR/8/34(H1N1) Virus.

Compound	Assay	CC_50_ [μM]	EC_50_ [μM]	SI
EGCG	Plaque formation reduction	275.5 ± 6.00 μM	94.6 ± 11.1 μM	2.91
EGCG-C18		300.0 ± 25.0 μM	64.0 ± 0.50 μM	4.68
EGCG-C18DE		250.0 ± 25.0 μM	7.00 ± 0.50 μM	35.7
EGCG-C18TE		32.0 ± 3.50 μM	3.00 ± 0.50 μM	10.6

**Table 7:** Cytotoxicity and Direct Virucidal Effect of EGCG and EGCG-Fatty Acid Derivatives against Influenza A/PR/8/34(H1N1) Virus.

Compound	Assay	CC_50_ [μM]	EC_50_ [μM]	SI
EGCG	Plaque formation reduction	275.5 ± 6.00 μM	0.391 ± 0.056 μM	703
EGCG-C18		300.0 ± 25.0 μM	0.060 ± 0.010 μM	5000
EGCG-C18DE		250.0 ± 25.0 μM	0.180 ± 0.050 μM	1389
EGCG-C18TE		32.0 ± 3.50 μM	0.10 ± 0.208 μM	320

CC_50_: The CC_50_ represents the concentration of compound required to reduce cell viability by 50% relative to the control well without test compound. EC_50_: The EC_50_ represents the concentration of compound required to reduce plaque number by 50% relative to the control well without test compound. SI: Selectivity index (SI) is the ratio of CC_50_ to EC_50_.

## Abbreviations

AAPH	2,2′-azobis(2-amidinopropane) dihydrocholoride
AMVN	2,2′-azobis (2,4-dimethylvaleronitrile)
bEGCdG	2′,2′-bisepigallocatechin digallate
BVDV	bovine viral diarrhea virus
C	(−)-catechin
CC_50_	50% cytotoxic concentration
CG	(−) catechin-3-*O*-gallate
CHIKV	chikungunya virus
DENV	dengue virus
DO-EGCG	5,7-dideoxy-EGCG
E	envelope protein
EBOV	Ebola virus
EC	(−) epicatechin
EC_50_	50% effective concentration
ECG	(−) epicatechin-3-*O*-gallate
EGC	(−)-epigallocatechin
EGCDG	epigallocatechin 3,5-digallate
EGCG	(−)-epigallocatechin-3-*O*-gallate
ELISA	enzyme-linked immunosorbent assay
EM	electron microscopy
GC	(−)-gallocatechin
GCG	gallocatechin-3-*O*-gallate
HA	hemagglutinin
HBV	hepatitis B virus
HCV	hepatitis C virus
HCVcc	cell-culture-derived HCV
HPV	human papilloma virus
HSV	herpes simplex virus
HVEM	herpes virus entry mediator
IAV	influenza A virus
IC_50_	50% inhibitory concentration
JEV	Japanese encephalitis
M	matrix protein
MALDI	matrix assisted laser desorption/ionization
MDCK	Madin-Darby canine kidney
MUNANA	2′-(4-methylumbelliferyl)-α-d-*N*-acetylneuraminic acid
NA	neuraminidase
NS	nonstructural protein
NMR	nuclear magnetic resonance
PA	polymerase subunit A
PB1	polymerase subunit B1
PB2	polymerase subunit B2
PRRSV	porcine reproductive and respiratory syndrome virus
RNP	ribonucleoprotein
RT	reverse transcription
SINV	sindbis virus
SEVI	semen-derived enhancer of virus infection
TCID_50_	50% tissue culture infectious dose
TF	theaflavin
TFDG	theaflavin-3,3′-*O*-digallate
TF-3-G	theaflavin-3-gallate
TF-3′-G	theaflavin-3′-gallate
WNV	West Nile viruses
YFV	yellow fever virus
ZIKV	zika virus
